# A Novel mHealth Approach for a Patient-Centered Medication and Health Management System in Taiwan: Pilot Study

**DOI:** 10.2196/mhealth.9987

**Published:** 2018-07-03

**Authors:** Wen-Ting Hsieh, Yung-Cheng Su, Hsin-Lien Han, Ming-Yuan Huang

**Affiliations:** ^1^ Biomedical Research and Innovation Incubation Center MacKay Memorial Hospital Taipei Taiwan; ^2^ Department of Emergency Dalin Tzu Chi Hospital Buddhist Tzu Chi Medical Foundation Chiayi Taiwan; ^3^ School of Medicine Tzu Chi University Hualien Taiwan; ^4^ Department of Medicine MacKay Medical College New Taipei City Taiwan; ^5^ Department of Emergency MacKay Memorial Hospital Taipei Taiwan

**Keywords:** mHealth, mobile app, QR code, medication management, medication adherence

## Abstract

**Background:**

Mobile health (mHealth) apps have recently demonstrated the potential to engage and empower people to improve their own health. Although the availability of health-related apps is increasing, their adoption rate in Taiwan is exceptionally low mainly due to the preponderance of Western culture-based app designs that are challenging for non-English-speaking individuals. To our knowledge, no mHealth app is available in Taiwan that is culturally tailored for Chinese-speaking users and that applies a patient-centered approach to self-manage medication and health.

**Objective:**

The purpose of this study was to design and deploy a culturally tailored mHealth system that could be easily integrated into current clinical practice and to evaluate how this mHealth system could support the continuity of patient care in Taiwan.

**Methods:**

An mHealth information system and a mobile app were designed. To promote the best patient experience, a Quick Response (QR) code system was developed to enable efficient registration of personal medication information through the mobile app. The app also supported notifications for drug utilization, refills, and symptom checks. Patients were encouraged to record medication use, symptoms, and self-assessments in the app during their treatment period. Evaluation of the novel mHealth system was conducted from August 1, 2016 to December 31, 2016 at MacKay Memorial Hospital, Taipei, Taiwan. Population data and app usage statistics were analyzed.

**Results:**

During the 5-month implementation period, a total of 25,909 users downloaded the app with an overall 7-day retention rate of 15.4% (SD 3.9). Young male adults (range 25-44 years) were the predominant user population. Patients’ feedback on app usability and design, QR code system as drug input method, medication reminders, and linking family or friends into care networks was generally positive. Physicians showed great interest in utilizing patient-generated data in their care process, and the positive medication adherence rate was the most highly valued component of this system.

**Conclusions:**

This pilot study demonstrated the value of a novel mHealth approach for individualized medication and health management in Taiwan. The mHealth system shows the potential to optimize personalized care into existing clinical services and may help hospitals and health authorities perform continuous quality improvement and policy development.

## Introduction

In 2003, the World Health Organization (WHO) reported that medication nonadherence was a global concern, especially for long-term therapies and resulted in increased morbidity [1], mortality [2], and unnecessary medical expenditure [3]. In Taiwan, unnecessary medical expenditure caused by medication nonadherence is a serious problem. A 2016 statistical report of the Taiwan National Health Insurance Administration stated that the average number of prescribed drugs per prescription of each patient in Taiwan was 3.16, which was higher than that in Western countries [4]. Approximately 25% of prescribed drugs are not taken by patients (costing at least NT $30 billion), and 2.6 tons of unused and expired drugs are discarded annually [5]. These phenomena indicate that solving the problem of medication nonadherence in Taiwan is a critical issue.

Medication nonadherence may be caused by patients’ intentional or unintentional behaviors. Intentional nonadherence refers to deciding not to take a medication based on the patient’s own perceptions [6-8]. For instance, incomplete medication knowledge may result in the fear of adverse side effects and is often the intentional reason for medication nonadherence. In contrast, unintentional nonadherence means that the patient intends to take a medication as prescribed but fails to do so because of forgetfulness or carelessness. Patients’ demographic and clinical characteristics, complicated regimens associated with polypharmacy, and patient-physician interaction may cause unintentional medication nonadherence [6-10]. For years, many interventions such as reminders, counseling, reinforcement, or education have been used to improve medication adherence by changing patients’ behaviors [11,12]. Awareness and appropriate selection the elements of intentional and nonintentional determinants for the target population are necessary for the design and development of tailored solutions for medication adherence.

Recently, the popularization of internet technology and mobile health (mHealth) tools for public health or medical care purposes have transformed human life significantly [13,14]. Short message service text messages, sent by mobile phones using reminder systems, have produced positive effects on medication adherence in patients with chronic diseases and those requiring health care services [15-17]. Studies indicate that mHealth apps have been widely applied in the medical management of patients with cancer [18], diabetes [19,20], cardiovascular disease [21,22], and other chronic diseases [23]. Although many health-related apps are available in the market, the use of these apps in Taiwan is low because most apps are designed based on Western cultures. For instance, many medication apps, including MediSafe (Medisafe Project Ltd), DoseCast (Montuno Software, LLC) and MyMeds (MyMeds, Inc), aim to help patients improve their medication adherence [24]. However, the English user interface of these apps poses a challenge for non-English speaking users. The language barrier makes it difficult for Chinese people to identify English drug names, thus reducing their willingness to better understand personal medications [25]. Also from a culture perspective, Chinese patients less frequently use somatic symptom descriptions, compared with Western patients, and instead use Yin-Yang energy balance to discuss illness [26]. Compared with Western patients, Chinese patients normally include family members as important influencers in medical decision-making processes [26]. To our knowledge, no app is available in Taiwan that is culturally tailored for Chinese-speaking users and which provides a patient-centered approach for personal health self-management. The current mHealth Apps in Taiwan are mainly used for operational purposes, such as appointment scheduling, medication refill notification, patient queue monitoring, and mobile payment [27].

The purpose of this study was to deploy a novel mHealth system that could help Chinese-speaking patients to self-manage their medication and health and to understand how this mHealth system could support the continuity of patient care in Taiwan. The new system leverages cloud technologies to integrate with existing hospital information systems and applies a patient-centered design principle for culturally tailoring to Chinese people, with easy-to-use medication registration, symptom tracking, drug information review, and quick health self-assessment. This system also aims to facilitate the coordination of care by seamless information sharing among patients and families.

## Methods

### System Design and Informational Framework of the mHealth Solution

**Figure 1:**
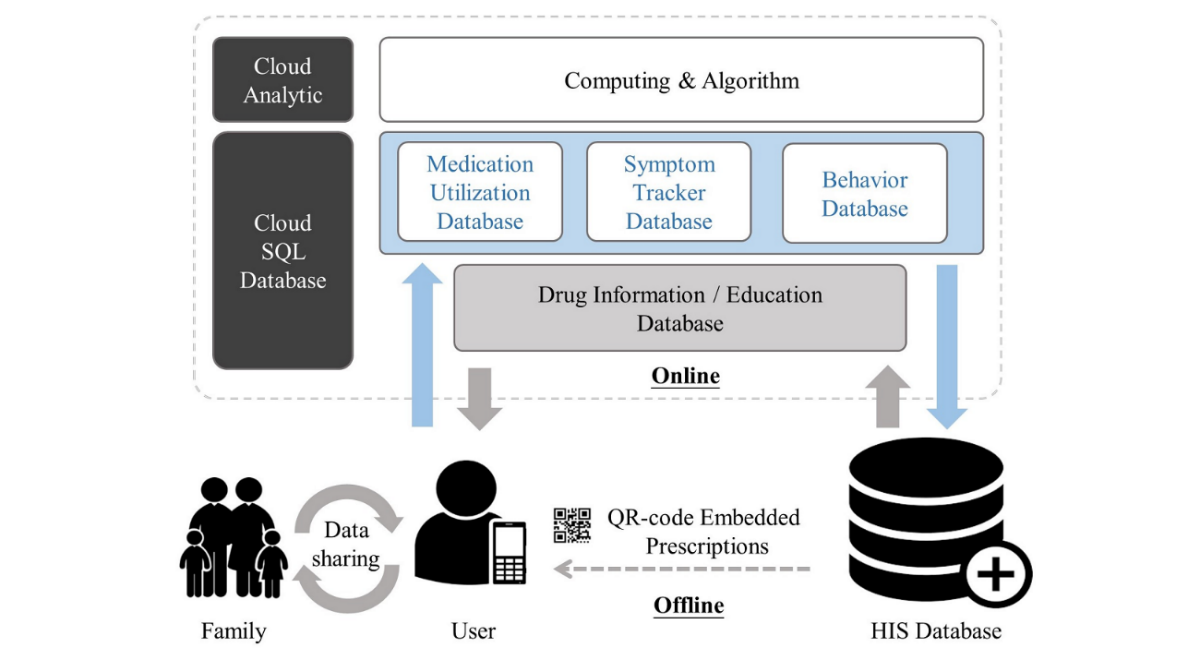
Systemic architecture for the mHealth system. HIS: hospital information system, QR: Quick Response, SQL: structured query language.

The mobile app was developed cooperatively by MacKay Memorial Hospital (MMH), Taiwan, and HTC Corporation, Taiwan, for use on iOS and Android platforms. The information structure and design of the mHealth system is illustrated in Figure 1. A simple Quick Response (QR) code system was designed specifically to interoperate our mHealth cloud system with the current hospital information system (HIS) in Taiwan. Our medical prescription notes and drug packages for patients routinely carry the information and posology of the prescribed drugs. We integrated all this information into a QR code and incorporated it into the present prescription notes and drug packaging system so that personal medications could be easily registered in the mobile app by simply scanning QR codes in an offline environment. More information about prescription drugs, including photos, side effects, and interaction cautions can be synchronized with the hospital drug database, as long as the user’s mobile phone is online. This 2-step operational design allowed rapid placement of new mHealth systems, to comply with national safety and security regulations, without the need to adjust existing HIS architecture. All usage data, including medication utilization, symptoms, and drug-related behaviors, are stored in the cloud structured query language (SQL) database. After registering the medications, users were notified when to take each medication. In addition to self-managing medications, users can also share their actual medication utilization status with family members and physicians.

### Introduction to the Mobile App for Personal Medication Management

**Figure 2:**
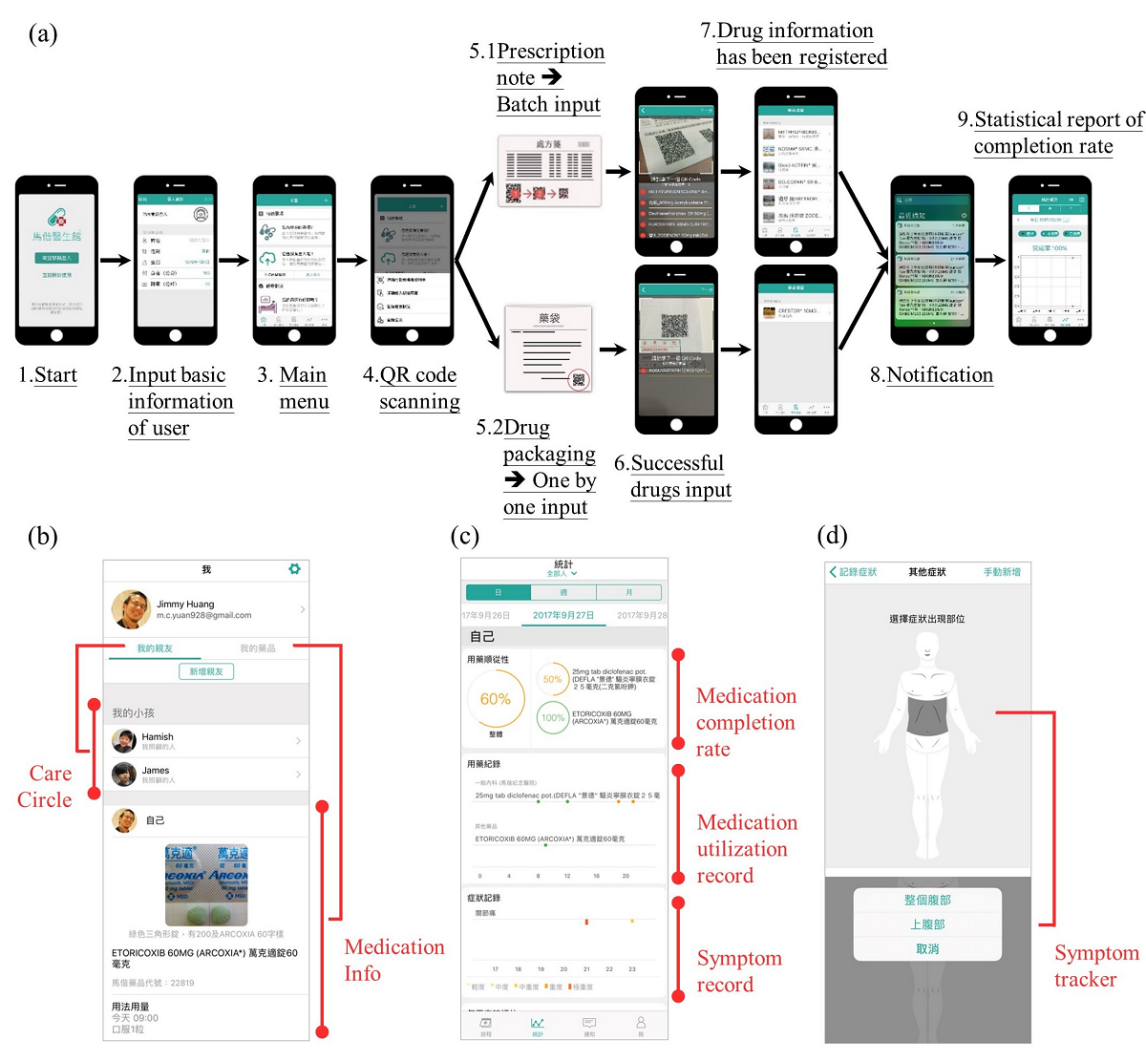
Screenshots of the (a) operation procedures for medication entry by the Quick Response (QR) code system, (b) care circle feature, (c) user medication completion reports, and (d) symptom checker of the app.

The app features 3 main functions tailored for Chinese culture that cover several determinants of medication adherence: (a) medication self-management, (b) care circle, and (c) symptom records and global self-assessment. The app operation procedure is illustrated in detail in Figure 2a. At MMH, prescription notes for chronic diseases and all drug packaging are printed with QR codes that contain personal drug information, including the drug name, prescribing physician’s name, and dosage frequency and duration of every drug. After scanning the QR code with the app, all the above-mentioned information plus detailed medication information in the Chinese language will be immediately transmitted to the users’ mobile phones, and users can share their data with their family or friends for mutual support and better engagement, which may help to reduce the intentional determinants of medication nonadherence (Figure 2b). To further eliminate the unintentional determinants of medication adherence, the app also provided a personal notification function, according to the original prescription, as a medication reminder to each user. For a patient’s medication self-management, the system will establish daily, weekly, and monthly statistical reports of each patient’s completion rates by analyzing their medication utilization records (Figure 2c). In addition, users were encouraged to record their symptoms or discomforts, as well as global self-assessment, through a user-friendly interface design (Figure 2d). Global self-assessment enables users to quickly determine their degree of current treatment outcomes using a 5-grade Likert scale as a quantitative rating.

### Implementation of the Pilot Study

The deployment process of the system consisted of different channels and activities in the hospital from August 1, 2016 to December 31, 2016. First, for general users, the app was initially launched through iOS and Google Play stores. However, we realized that patients were the major target audience in this pilot study, and several promotional materials, including posters, flyers, and videos, were widely distributed within MMH. Help desks were also set up at several specific locations in the hospital, such as the pharmacy and reception counter, where in-house staff and project-recruited volunteers were able to interact with patients and help them download and use the app. Second, we promoted the system in some consultation rooms and partnered with medical care providers to help deploy the app at outpatient clinics. In this pilot study period, we selected cardiology, cardiovascular surgery, and rheumatology clinics as our target groups. The patients were encouraged to use the app by their primary care physicians so that the physicians could understand the patients’ medication usage reports on their return visits. Third, for hospitalized patients from internal medicine wards, instruction manuals and consultations were provided before discharge so that they could then self-manage their medications and to better bridge their care from in-hospital to outpatient status.

### Data Analysis

#### Collected Measures

Data of all participants from the pilot study were recorded by computational backend. In our cloud, the SQL database used a python framework (3.0) to automatically retrieve predefined information from the cloud database. We collected the behavior, number, age, and gender of users, as well as the numbers of drugs by scanning prescription note (batch) or drug packaging (single) and count by browsing the information for each drug. The 7-day active users were defined as those users who used the app any time during the previous 7 days. Data were automatically collected every week. The 7-day retention rate was defined as the percentage of unique users who were still using the app for 7 consecutive days after the first installation.

#### Analyses

IBM SPSS Statistics version 19 (IBM Corp, Armonk, NY, USA) was used to analyze the data. The proportion of each scanned drug was defined as the scanning counts of each drug divided by the total scanning counts of all drugs. The completion rate was defined as the doses of each medication that were taken by the user divided by those of each medication prescribed by the physician. The number of 7-day active users, number of daily clicks per user, and 7-day retention rate are presented as mean (SD). The data for age, gender, and drugs were only accessible from the Android platform. Feedback from the users of the system, including Google star rating and comments, were also assessed. Feedback taken by convenience sampling at outpatient clinics from the physicians was also collected for suggestions and future collaboration.

### Ethics Approval

This study was approved by the Institutional Review Board of Taipei Mackay Memorial Hospital, Taiwan (No.18MMHIS016e).

## Results

### Evaluation of User Behaviors

**Figure 3:**
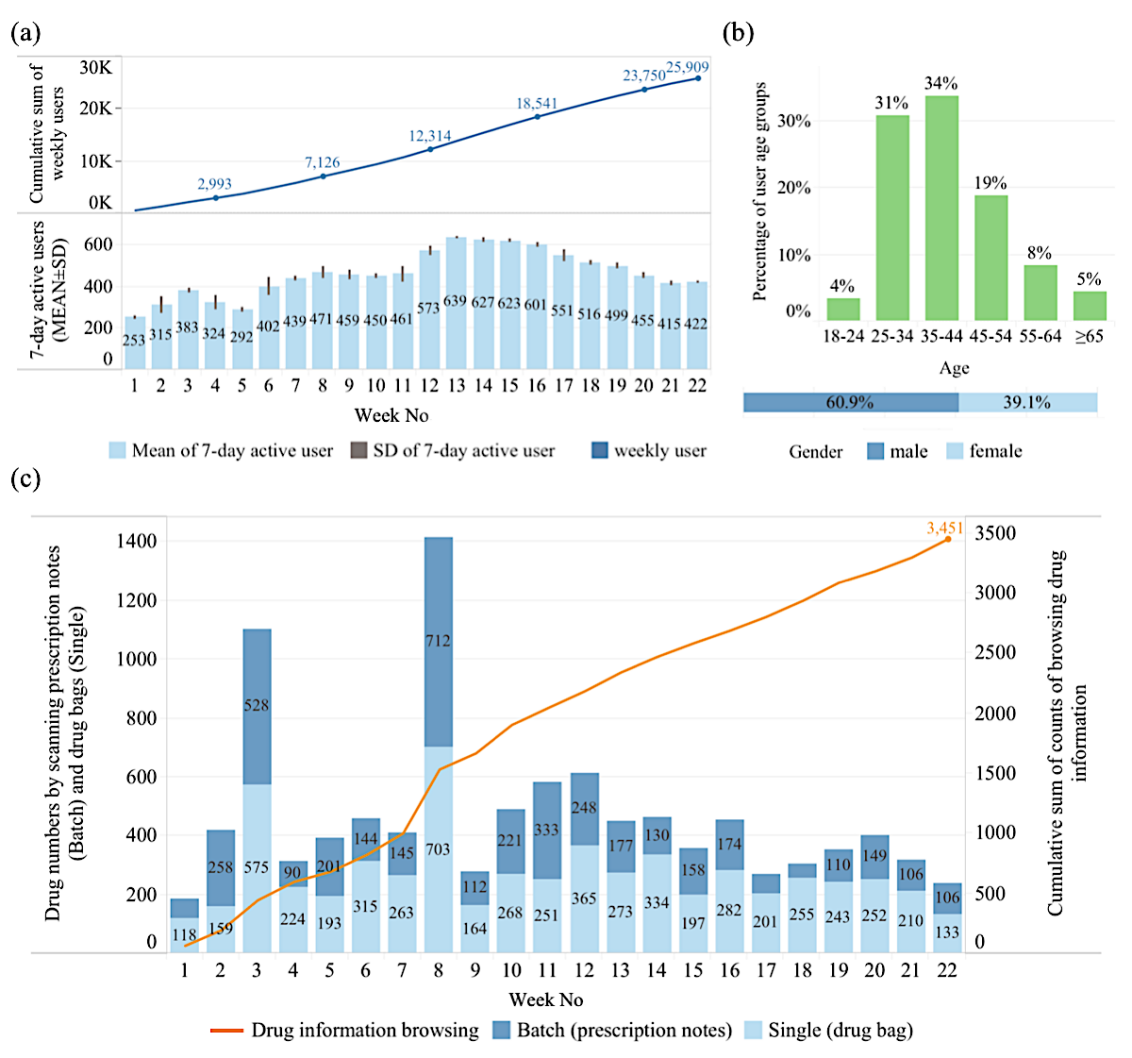
(a) The cumulative user number and average of 7-day active users, (b) age and gender demographics, and (c) the cumulative count of browsing drug information and the number of medication registration by scanning prescription notes or drug packaging during the 5-month pilot period illustrated by 7-day interval (week).

The pilot test for evaluating the new mHealth system was initially conducted at MMH from August 1, 2016 to December 31, 2016. A total of 25,909 users downloaded the app, and the mean of 7-day active users over a 7-day interval during the implementation period ranged from a minimum of 253 (8; week 1, Aug 1-7) to a maximum of 638 (7; week 13, Oct 24-30) people, with increasing numbers of users every week since implementation (Figure 3a). In addition to one underage person (age<18 years) who was not allowed to register the app, the age distribution of the total user population was divided into 6 groups, including 18-24, 25-34, 35-44, 45-54, 55-64, and >65 years old. The percentages of age and gender distribution were showed in Figure 3b. The demographic results demonstrated that younger adults (range 25-44 years), especially males, were the highest user population that engaged in the study in the 5-month implementation period at MMH.

The ways of registering medications were also investigated. In the app system design, users could register their drugs by scanning the QR code on the prescription note or drug packaging. As shown in Figure 3c, based on statistical analysis of the cumulative drug numbers scanned from the drug packaging, the numbers ranged from a minimum of 118 (week 1, Aug 1-7) to a maximum of 703 (week 8, Sep 19-25). Based on the scanning of prescription notes, the cumulative drug numbers ranged from a minimum of 48 (week 18, Nov 28 to Dec 4) to a maximum of 712 (week 8, Sep 19-25). Thus, during the implementation period, the number of drugs scanned from drug packaging (single input, green bar) was higher than that from the prescription notes (batch input, blue bar). These results indicated that the users preferred to scan the drug packaging as a means of registering their medications in the app, rather than using the prescription notes.

Statistical analysis of browsing the drug screen, which included drug pictures and detailed information, was used to initially assess whether the system could help users to enhance their knowledge of medications. Medication information surfing behavior via the app gradually increased over time, reaching 3445 times at the fifth month of the study (Figure 3c), which suggested that more patients were familiar with the system as a channel through which they could acquire their medication knowledge. Of the 25,909 users, a 7-day retention rate of 15.4% (SD 3.9) was observed during the implementation period. Among daily active users, the counts of browsing the drug screen per person, 0.13 (SD 0.15) times (data not shown), were much less than daily total clicks in the app per person, 30.6 (SD 5.8) times.

### Evaluation of Feedback

The Google star rating of the app was 4.493 points out of 5, over a total of 64 reviews, and some of the comments from the users are as follows:

It was very convenient that I could manage all the drugs by scanning the QR codes on the prescription note or drug packaging and was notified for each scheduled drug without forgetting to take each medication.

The App was a useful tool for me because I could inquire about the function of my drugs and record my symptoms when I was uncomfortable.

Feedback taken by convenience sampling from physicians at hospital outpatient clinics was mostly positive. Physicians generally showed great interest in seeing the patients’ medication usage reports for the first time, including medication completion rate, symptoms, and global self-assessment of the patient at every return visit (formats of reports are shown in step 9 of Figure 2a-c).

It does make me feel comfortable when my patient tells me that he uses this software to remind him to take medicine. And I know that the patient is engaged and motivated by the intervention.

Seeing the patient in my clinic showing me the software with the number of his personal medication completion and symptom status really make me understand my patients better.

### Evaluation of Drug Registration

**Figure 4:**
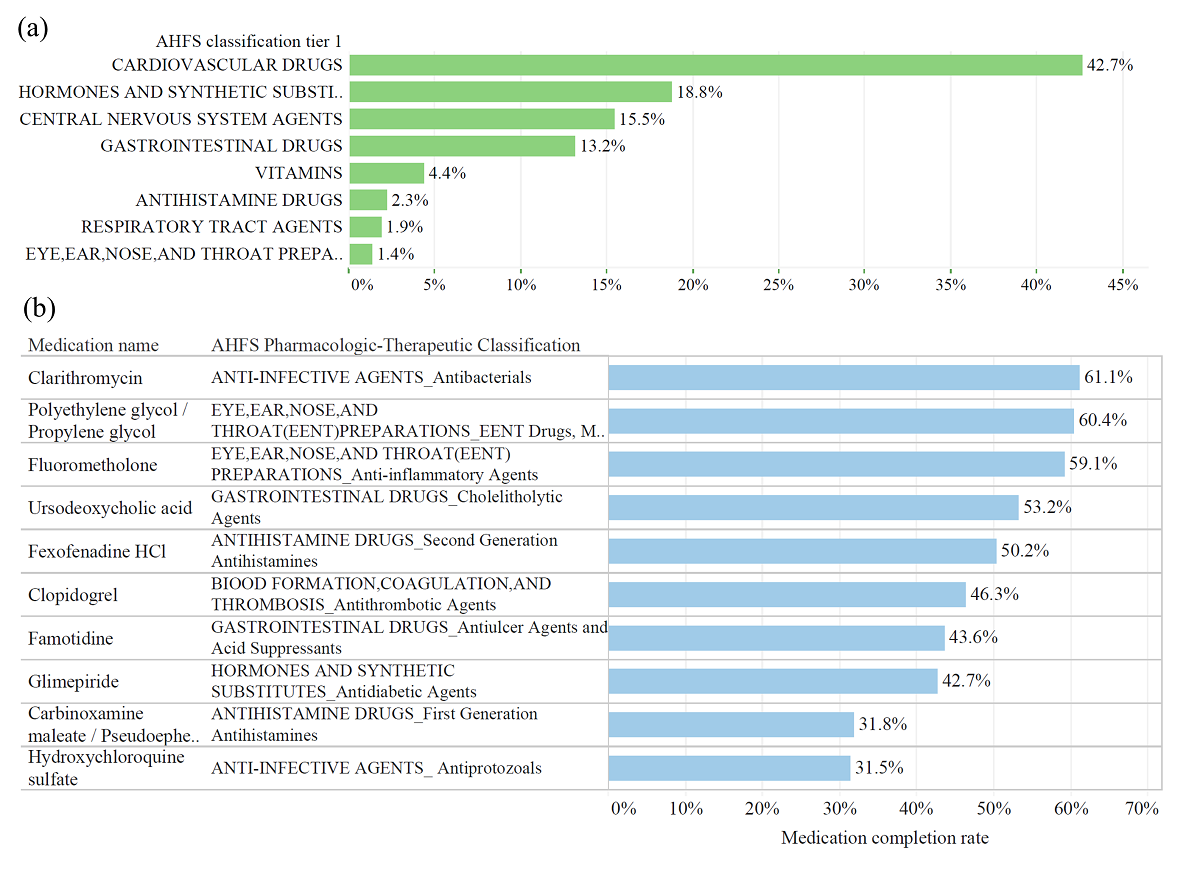
(a) Categories of top 30 registered medications and (b) the top 10 drugs ranked by completion rate from the mHealth system in 5-month pilot period.

The counts of total medication accessed by scanning the prescription notes or drug packaging were extracted from computational backend. A total of 25,267 scanning counts were analyzed using first-tier and second-tier drug categories based on the American Hospital Formulary Service classification, 2017 edition, and the top 30 registered drugs are listed in Multimedia Appendix 1. Drugs for chronic diseases, including cardiovascular drugs (42.7%), hormones and synthetic substitutes (18.8%), and central nervous system drugs (15.5%), represented the three major drug categories among the top registered drugs of the system (Figure 4a).

The medication usage status could be also tracked and analyzed by computational backend, and the top 10 drugs ranked by completion rate are shown in Figure 4b and Multimedia Appendix 2. Drugs for anti-infective agents, ophthalmic preparations, gastrointestinal drugs, and antiallergic agents showed the highest level of medication adherence, with completion rates greater than 50%. It is interesting that, although the chronic disease medication was most highly registered, the completion rate was low.

## Discussion

### Principal Findings

As the population ages and cost pressures increases, the health care industry continues to face challenges and must find ways to enhance the role of patients in the management of their diseases. One of the ways by which patients can better manage their disease is to adhere to their medication regimens. This pilot study aimed to examine how to make patients familiar with using a new mHealth app for their medical treatments and to investigate the efficacy of integrating an mHealth system into clinical practice at a Taiwan hospital. Our experience suggests that the novel mHealth approach for personal medication and health management in Taiwan is feasible and that the mobile solution incorporating a QR code system and an innovative 2-step system design can provide an integrative solution for other medical institutions in Taiwan as a digital tool for engaging patients to encourage self-management.

Although many users were recruited during the implementation period, we found that the 7-day retention rate of the new mHealth app was low, 15.4% (SD 3.9), compared with previous randomized controlled trials for medication adherence [28,29]. This may be because our app was initially launched to the public as a free download to participate in the pilot study, rather than to perform a rigorous clinical trial for a specific population. The finding is also consistent with previous studies showing that frequent use of the app declined substantially within the first 2 months [30,31]. The high attrition rate for this intervention may reflect users’ interest in the novelty of the app, which declined rapidly as the novelty disappeared. The low 7-day retention rate implies that human factors play an important role in developing a better patient-centered mHealth app for better retention. Also, awareness of the digital health experience was still low among users in Taiwan, and more efforts are needed to establish greater awareness and use of mHealth services.

In addition, the differences between daily counts of browsing the drug screen per person, 0.13 (SD 0.15) times, and daily clicks in the app per person, 30.6 (SD 5.8) times, demonstrated that the users preferred to use notifications, symptom records, global self-assessments, and statistical reports of completion rates rather than the medication information. This suggests that it is imperative we strengthen patients’ knowledge of medication in Taiwan.

In this study, younger adult males (range 25-44 years) were the predominant population who volunteered to engage with the app. This finding is consistent with previous studies [30,32-34] and demonstrates that younger adults are more likely to accept mobile devices. Simultaneously, this finding also suggests that a “digital divide” continues to exist between the genders, and males are more engaged in using mobile technology than females. Our findings also indicate that the demographic characteristics of mobile technology in the United States and Germany, as well as in Taiwan, follow similar trends [34,35]. Although elderly patients (>65 years) with chronic diseases are typically considered to be a population who often needs additional tools to address low medication adherence because of their complicated comorbidities and polypharmacy, we observed that in this pilot study, few elderly patients used the mHealth app in Taiwan. Interestingly, previous studies demonstrated differences between older adults and younger adults in their perceptions, preferences, and adoption of mobile technology [30,36,37]. The “Ambient Assisted Living Project’’ reported that the older group of patients had very high acceptance of 7-inch tablet computers used as a medication management app [30,38]. Applying appropriate assistive devices may be as important as developing well-designed mobile apps for establishing an mHealth strategy for elderly patients.

Historically, several methods for measuring medication adherence, such as pill counts, refill rates, patient self-reports, biological and electronic monitoring, have been proposed [39-41]. In this study, the Taiwanese population-based medication usage behavior could be timely tracked for the first time, and the results highlighted that the adherence to medications for chronic diseases was relatively low. This objective data has the potential to provide reference values for quality improvement measures and policy development by hospital and government administrations. Another advantage of the new mHealth system is that it allowed our patients to be able to report the objective measures of their medication adherence to health care providers. However, results of this pilot study also showed fragmented completion rates of drug usage, suggesting that even though users were notified by the system, patients continued to forget to take medications and some never even used the app. In addition to medication administration reminders for patients, an alarm system may be needed to integrate with current care circle design to ensure patients’ medication intake by instantly alerting the members of the care circle when the system detects that the patient was not taking medication. Our results also suggest that the next stage for improving the new mHealth system is to understand how to engage Taiwanese patients to continue reporting their medication utilization status and outcome measures.

### Limitations and Solutions

During the 5-month implementation period, the pilot project showed certain limitations worth discussing and resolving. First, demographic results, including age and gender as well as the drug categories ranked by completion rate, were only extracted from the Android platform because our computational backend had no access to extract the data from the iOS platform. We tried to analyze the statistical numbers collected from the two platforms separately and found that there was no significant difference between the platforms, suggesting that the two populations showed similar usage behaviors. However, additional technical support is still needed to solve this problem in continuing updates of the new mHealth app.

Second, the patients tended to use the system in a repetitive way (ie, drug packaging, single input) to register each drug instead of registering all drugs just once (ie, prescription notes, batch input). However, the QR codes were unclear because of blurry prints, and creases caused medication registration in the app to fail at a relatively high rate during the implementation period. Therefore, QR codes must be printed on the prescription notes not only for chronic diseases but also for other diseases, and more promotion activities must be implemented to assist patients. By encouraging patients to register their medications through the prescription notes, we hope to avoid wasting time and reduce registration failures.

Third, in the predefined data retrieval, we only defined clicks either from browsing the drug screen or from other information check, including drug notification, symptom records, global self-assessment, and statistical reports of completion. Under this circumstance, we are unable to provide specific data for each function usage. We also could evaluate different periods of retention rate, other than 7 days, in our current system. For example, it would be interesting to know whether the 30-day retention rate is even lower to determine if patients would not use the app over a long term for medication compliance. Furthermore, since we did not retrieve users’ information from the cloud database, we were unable to know the sources of users. As a result, we cannot analyze if the behaviors are different among users from the public, clinics, or hospitalization.

Finally, although the physicians could search patients’ medication usage reports and symptoms from the smartphone at every return visit, this additional action still increases physician consultation time. Therefore, the collaborative patient-physician interaction needs to be optimized by building an encrypted Web-based information system so that physicians can directly access the patients’ medication reports and symptoms by using HIS, thereby reducing physician consultation time.

### Future Developments

We believe that the promising achievements of this project will lead to further improvements for the innovative mHealth system. First, we will investigate the unmet needs of patients and physicians in their collaborative interaction and continually expand functions of the app to further optimize the newly developed patient-centered mHealth system. Second, we plan to execute a clinical trial to validate whether the mHealth app can significantly improve patients’ medication adherence after we receive informed consent from patients, following approval of the institutional review board at MMH.

Understanding the benefits of the system, physicians from different specialties, such as cardiovascular surgeons and pediatric rheumatologists, were especially interested in this system as their patients were among those with the most chronic conditions, involving multiple and complicated medications, with typically difficult self-management. The statistical numbers from the active users within the 5-month period indicated that the mHealth app would be a potential health care tool for Taiwanese patients. Moreover, we plan to provide tailored mHealth solutions for other physicians with different specialties and to help patients with different diseases have better therapeutic outcomes. Finally, we believe that the quality and design of our digital health solution for personal medication and health management can be easily integrated into other hospitals and will be the foundation that allows health care institutions to provide a more versatile and personalized approach toward advanced health care.

### Conclusion

This pilot study investigated the role of a culturally tailored mHealth system for personal medication and health management in Taiwan. Study results show that the app’s medication registration and notification system helped users self-manage their complicated polypharmacy regimens. The most important feature of the new patient-centered mHealth system was that it reflected patients’ actual medication utilization status, symptoms, and self-assessment. The information is readily available to their physicians through optimization of the patient-physician digital experience so that health care providers and patients can function in a collaborative manner and facilitate patients’ medication adherence. The innovative mHealth solution has the potential to understand Chinese-speaking patients’ medication adherence and therapeutic outcomes as well as to engage patients and family as partners in long-term medical care.

## Appendix

Multimedia Appendix 1 The top 30 registered drugs by the mHealth system.

Multimedia Appendix 2 Top 10 drugs ranked by completion rate.

## Abbreviations

HIS: hospital information system
mHealth: mobile health
MMH: MacKay Memorial Hospital
QR: Quick Response
SQL: structured query language
WHO: World Health Organization
