# Correction: A Novel mHealth Approach for a Patient-Centered Medication and Health Management System in Taiwan: Pilot Study

**DOI:** 10.2196/27292

**Published:** 2022-02-15

**Authors:** Wen-Ting Hsieh, Yung-Cheng Su, Hsin-Lien Han, Ming-Yuan Huang

**Affiliations:** 1 Biomedical Research and Innovation Incubation Center MacKay Memorial Hospital Taipei Taiwan; 2 Department of Emergency Dalin Tzu Chi Hospital Buddhist Tzu Chi Medical Foundation Chiayi Taiwan; 3 School of Medicine Tzu Chi University Hualien Taiwan; 4 Department of Medicine MacKay Medical College New Taipei City Taiwan; 5 Department of Emergency MacKay Memorial Hospital Taipei Taiwan

In “A Novel mHealth Approach for a Patient-Centered Medication and Health Management System in Taiwan: Pilot Study” (JMIR Mhealth Uhealth 2018;6(7):e154) one addition was made.

In the Methods section of the originally published paper, the subsection “Ethics Approval” has been added, containing the following statement:

This study was approved by the Institutional Review Board of Taipei Mackay Memorial Hospital, Taiwan (No.18MMHIS016e).

The correction will appear in the online version of the paper on the JMIR Publications website on February 15, 2022, together with the publication of this correction notice. Because this was made after submission to PubMed, PubMed Central, and other full-text repositories, the corrected article has also been resubmitted to those repositories.

