# Supplementary material for: A Novel mHealth Approach for a Patient-Centered Medication and Health Management System in Taiwan: Pilot Study
Source: JMIR Mhealth Uhealth. 2018 Jul 3;6(7):e154. doi: 10.2196/mhealth.9987 (PMC6053609; doi:10.2196/mhealth.9987)
Supplement: Multimedia Appendix 1 [file mhealth_v6i7e154_app1.pdf]

**Table 1.** Top 30 drugs ranked by scanning counts.

| Rank | Drug (Generic name) | AHFS pharmacologic category (Classification first tier) | AHFS pharmacologic category (Classification second tier) | Proportion (scanning counts) [a] |
|---|---|---|---|---|
| 1 | Propranolol | Cardiovascular Drugs | Cardiac Drugs | 6.25% (1579) |
| 2 | Alprazolam | Central Nervous System Agents | Anxiolytics, Sedatives and Hypnotics | 5.16% (1303) |
| 3 | Glimepiride | Hormones and Synthetic Substitutes | Antidiabetic Agents | 4.76% (1202) |
| 4 | Rosuvastatin | Cardiovascular Drugs | Antilipemic Agents | 4.53% (1144) |
| 5 | Valsartan | Cardiovascular Drugs | Hypotensive Agents | 4.31% (1088) |
| 6 | Vildagliptin/ Metformin | Hormones and Synthetic Substitutes | Antidiabetic Agents | 4.04% (1022) |
| 7 | Bisoprolol fumarate | Cardiovascular Drugs | Hypotensive Agents | 3.35% (847) |
| 8 | Fursultiamine (TTFD)/ Vitamin B2 | Vitamins | Vitamin B Complex | 3.27% (825) |
| 9 | Metformin HCl | Hormones and Synthetic Substitutes | Antidiabetic Agents | 3.19% (808) |
| 10 | Bisoprolol fumarate | Cardiovascular Drugs | Hypotensive Agents | 2.86% (723) |
| 11 | Famotidine | Gastrointestinal Drugs | Antiulcer Agents and Acid Suppressants | 2.58% (651) |
| 12 | Acetaminophen | Central Nervous System Agents | Analgesics and Antipyretics | 2.39% (604) |
| 13 | Fludiazepam | Central Nervous System Agents | Anxiolytics, Sedatives and Hypnotics | 2.18% (551) |
| 14 | Silymarin | Gastrointestinal Drugs | Lipotropic Agents | 2.16% (547) |
| 15 | Acarbose | Hormones and Synthetic Substitutes | Antidiabetic Agents | 2.03% (514) |
| 16 | Pitavastatin calcium | Cardiovascular Drugs | Antilipemic Agents | 1.88% (475) |
| 17 | Aspirin (Acetylsalicylic acid) | Central Nervous System Agents | Analgesics and Antipyretics | 1.83% (463) |
| 18 | Fexofenadine HCl | Antihistamine Drugs | Second Generation Antihistamines | 1.69% (426) |
| 19 | Amlodipine besylate | Cardiovascular Drugs | Hypotensive Agents | 1.67% (421) |
| 20 | Lercanidipine HCl | Cardiovascular Drugs | Hypotensive Agents | 1.62% (409) |
| 21 | Pravastatin | Cardiovascular Drugs | Antilipemic Agents | 1.53% (386) |

| | | | | |
|---|---|---|---|---|
| 22 | Amlodipine & Valsartan | Cardiovascular Drugs | Hypotensive Agents | 1.48% (374) |
| 23 | Domperidone | Gastrointestinal Drugs | Antiemetics | 1.43% (361) |
| 24 | Sodium cromoglycate nasal solution | Respiratory Tract Agents | Anti-inflammatory Agents | 1.41% (356) |
| 25 | Sennoside | Gastrointestinal Drugs | Cathartics and Laxatives | 1.35% (341) |
| 26 | Dimethylpolysiloxane | Gastrointestinal Drugs | Antiflatulents | 1.29% (327) |
| 27 | Nicorandil | Cardiovascular Drugs | Vasodilating Agents | 1.22% (309) |
| 28 | Dipyridamole | Cardiovascular Drugs | Vasodilating Agents | 1.22% (307) |
| 29 | Magnesium oxide | Gastrointestinal Drugs | Antacids and Adsorbents | 1.04% (263) |
| 30 | Neostigmine methylsulfate oph soln | Eye,Ear,Nose,and Throat(EENT)Preparations | Antiglaucoma Agents | 1.01% (256) |

[a] Total of 25,267 scan counts of all medication during the study period.
