# Supplementary material for: A Novel mHealth Approach for a Patient-Centered Medication and Health Management System in Taiwan: Pilot Study
Source: JMIR Mhealth Uhealth. 2018 Jul 3;6(7):e154. doi: 10.2196/mhealth.9987 (PMC6053609; doi:10.2196/mhealth.9987)
Supplement: Multimedia Appendix 2 [file mhealth_v6i7e154_app2.pdf]

Table 2. Top 10 drugs ranked by completion rate.

Rank	Drug (Generic name)	AHFS pharmacologic category (Classification first tier)	AHFS pharmacologic category (Classification second tier)	Completion rate [b]
1	Clarithromycin	Anti-infective Agents	Antibacterials	61.1%
2	Polyethylene glycol/ Propylene glycol	Eye,Ear,Nose,and Throat(EENT)Preparations	EENT Drugs, Miscellaneous	60.4%
3	Fluorometholone	Eye,Ear,Nose,and Throat(EENT)Preparations	Anti-inflammatory Agents	59.1%
4	Ursodeoxycholic acid	Gastrointestinal Drugs	Cholelitholytic Agents	53.2%
5	Fexofenadine HCl	Antihistamine Drugs	Second Generation Antihistamines	50.2%
6	Clopidogrel	Blood Formation, Coagulation,and Thrombosis	Antithrombotic Agents	46.3%
7	Famotidine	Gastrointestinal Drugs	Antiulcer Agents and Acid Suppressants	43.6%
8	Glimepiride	Hormones and Synthetic Substitutes	Antidiabetic Agents	42.7%
9	Carbinoxamine maleate/ Pseudoephedrine HCl	Antihistamine Drugs	First Generation Antihistamines	31.8%
10	Hydroxychloroquine sulfate	Anti-infective Agents	Antiprotozoals	31.5%

[b] The completion rate was calculated automatically by the system.
